# Energy-Dependent Effects of Pulsed Electric Field (PEF) Treatment on the Quality Attributes, Bioactive Compounds, and Microstructure of Red Bell Pepper

**DOI:** 10.3390/molecules31010088

**Published:** 2025-12-25

**Authors:** Katarzyna Rybak, Aleksandra Skarżyńska, Szymon Ossowski, Magdalena Dadan, Katarzyna Pobiega, Małgorzata Nowacka

**Affiliations:** 1 Department of Food Engineering and Process Management, Institute of Food Sciences, Warsaw University of Life Sciences-SGGW, Nowoursynowska 159c, 02-776 Warsaw, Poland; katarzyna_rybak@sggw.edu.pl (K.R.); aleksandra_skarzynska@sggw.edu.pl (A.S.); szymon_ossowski@sggw.edu.pl (S.O.); magdalena_dadan@sggw.edu.pl (M.D.); 2 Department of Food Biotechnology and Microbiology, Institute of Food Sciences, Warsaw University of Life Sciences-SGGW, Nowoursynowska 159c, 02-776 Warsaw, Poland; katarzyna_pobiega@sggw.edu.pl

**Keywords:** nonthermal food processing, electroporation, vitamin C, carotenoids, color, texture, structure, quality

## Abstract

This study evaluated the energy-dependent effects of pulsed electric field (PEF) treatment on the physicochemical properties, bioactive compounds, antioxidant activity, and microstructure of red bell pepper (*Capsicum annuum* L.). Red bell pepper tissue was treated at specific energy inputs ranging from 1 to 10 kJ/kg and compared with a fresh (untreated sample). The cell disintegration index (CDI) increased progressively with PEF energy, confirming enhanced membrane permeabilization and structural disruption. Structural analyses (SEM and micro-CT) confirmed the formation of pores and interconnected channels, particularly at moderate and high energies. PEF treatment caused a decrease in total polyphenols and flavonoids, whereas vitamin C and total carotenoid contents increased at intermediate energies. Antioxidant activity (ABTS, DPPH, FRAP) declined overall but remained at comparable levels for mild PEF exposure. A significant reduction in firmness was observed (from 17% to 27% compared with the untreated control), and color changes were dependent on the energy input, while microstructural degradation intensified as the energy level approached 10 kJ/kg. PEF treatment improved microbial stability, resulting in a measurable reduction in total viable counts and yeast and mold counts, particularly at higher energy inputs. FTIR, TGA, and NMR data confirmed molecular alterations without degradation of major components. Multivariate analysis (dendrogram, PCA) distinguished four characteristic response groups: fresh, low-energy (1–2 kJ/kg), moderate-energy (4–5 kJ/kg), and high-energy (10 kJ/kg). PEF treatment selectively modified red bell pepper tissue, enhancing permeabilization and carotenoid/vitamin C release while preserving visual quality at mild–moderate energies. These results demonstrate the potential of PEF as a nonthermal technique for tailoring the structural and functional properties of plant-based products.

## 1. Introduction

The fruits of peppers (*Capsicum annum* L.) can be classified as sweet or hot of different colors (mainly green, yellow, orange, red), which depend on the ability to synthesize chlorophylls and carotenoids, as well as the degree of ripeness. Sweet red bell pepper is a good source of micro- and macroelements, such as iron, zinc, potassium, phosphorus; vitamins, such as provitamin A, B_6_, C, and E; and different bioactive compounds, such as polyphenols (including the pungent capsaicinoids and flavonoids) and carotenoids (mainly capsanthin, capsorubin, zeaxanthin, β-carotene, β-cryptoxanthin and antheraxanthin) [1]. However, due to high water content, red bell pepper is perishable and susceptible to diseases; therefore, the annual postharvest losses are relatively high, almost 40% [2,3]. In order to counteract high losses of red bell pepper, different pretreatments and processing methods are implemented, e.g., ultrasound (US) [2,4,5], pulsed electric field (PEF) [3,4,5], ultraviolet C (UV) [3,6], ozone [3], cold plasma treatment [7], pulsed light (PL) [7], drying [5,7,8], etc.

Pulsed electric field is a nonthermal treatment, which has recently been used in food processing in order to achieve microbial inactivation, as well as enhance heat and mass transfer processes. During the treatment, solid material immersed in a liquid medium (usually tap water) is placed between two electrodes and affected by short-term, high-voltage pulses, which result in, among others, softening of the tissue and thus easier peeling, cutting, better extraction of bioactive components, and shortening of different unit operations. The impact of PEF on the tissue structure is based on the electroporation phenomenon, consisting of the formation of new or growth of existing pores in cell membranes under the influence of an external electric field exceeding critical electric field strength [3,9,10]. The electroporation can be temporary (reversible electroporation) or permanent (irreversible electroporation), depending on material properties (e.g., cell size and shape) and PEF parameters (electric field strength, pulse number and duration, shape of the pulse, frequency, etc.). The value of energy input during PEF is crucial to analyze. Generally, if a low electric field strength is applied, the reversible electroporation occurs, and vital functions and integrity of the cell are still maintained after the treatment. On the other hand, the application of high electric field strength causes permanent permeabilization of the membrane, leakage of intercellular content, and consequently cell death [8,10]. Irreversible electroporation is responsible for the enhancement of diffusion processes and inactivation of microorganisms [10]. The energy limit beyond which irreversible electroporation occurs is difficult to assess; therefore, estimation of cell disintegration index (CDI) is helpful to analyze the extent of electroporation [11]. CDI takes values in the range of 0 to 1, where 0 indicates an intact sample and 1 indicates a totally disintegrated sample [12]. The previous work investigated changes in the quality of red bell pepper after traditional and nonthermal treatment (PEF, US, US + PEF, PEF + US), revealed that a single PEF-treated red bell pepper was characterized by statistically unchanged carotenoids and vitamin C contents, as well as antioxidant activity in comparison with a fresh sample. Unlike blanching, which caused significant degradation of active substances [4]. However, in the articles, only a narrow range of energy was analyzed (up to 3 kJ/kg) with limited quality attributes.

Despite the growing interest in pulsed electric field (PEF) technology in plant-based foods, the existing literature is largely concentrated on low energy inputs applied to homogenized or liquid plant matrices, mainly aiming to improve extraction yields and juice processing efficiency [13]. Studies that systematically investigate the response of intact vegetable tissues across a wide range of PEF energy inputs remain limited. Most available reports do not integrate microstructural, mechanical, water mobility, and microbiological evaluations within a single experimental framework. This limits the mechanistic understanding of how electroporation-driven membrane permeabilization translates into macroscopic quality changes in whole vegetable tissues and restricts the rational optimization of PEF-assisted food processing.

This study was designed to test the hypothesis that increasing PEF energy input induces progressive electroporation and structural weakening of plant tissue, which, in turn, modifies color, texture, water mobility, microbial stability, and the extractability of bioactive compounds. It was further hypothesized that moderate energy levels would enhance the release of intracellular compounds without causing excessive tissue collapse, whereas excessive energy input would accelerate quality deterioration. Therefore, the aim of the study was to evaluate the effect of different PEF energy levels on the physicochemical, bioactive, antioxidant, and structural characteristics of red bell pepper, and to establish potential correlations among these parameters. The evaluated variables included cell disintegration index (CDI), color attributes (L*, a*, b*, ΔE), bioactive and antioxidant compounds (total polyphenols, flavonoids, carotenoids, vitamin C, ABTS, DPPH, FRAP), sugar profile (sucrose, glucose, fructose), structural and molecular properties (SEM, micro-CT, FTIR, NMR, TGA), and microbiological quality (TVC—total viable count; TYM—total yeast and mold).

## 2. Results and Discussion

### 2.1. Effect of PEF Treatment on the Physical Integrity of the Red Bell Pepper

#### 2.1.1. Cell Disintegration Index (CDI) of Red Bell Pepper Treated with PEF

**Figure 1:**
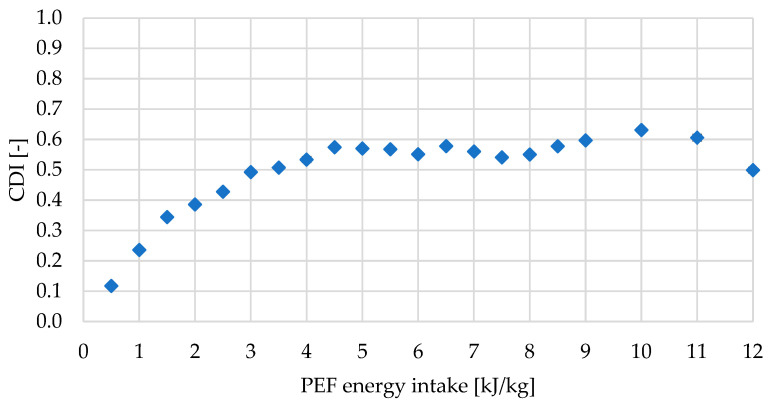
Cell Disintegration Index (CDI) of red bell pepper after PEF treatment with applied different energies.

The impact of PEF energy on the cell integrity of red bell pepper tissue was assessed by determining the Cell Disintegration Index (CDI) across a wide range of specific energy inputs (0–12 kJ/kg, in increments of 0.5 kJ/kg and later in 1 kJ/kg), as it was presented in Figure 1. The CDI reflects the degree of cell membrane permeabilization and disruption induced by the applied electric field [11].

In the current study, the maximum value of CDI was equal to 0.63 (for 10 kJ/kg), whereas in the study of Fauster et al. [14] for red bell pepper, it was up to around 0.8 for 6 kJ/kg. The differences can be due to the fact that the value of CDI is dependent on the type of material (link to size of cells, the content of fiber, etc.) and parameters of PEF (e.g., electric field strength, chamber configuration, or pulse number, amplitude, and shape). For instance, Fauster et al. [14] determined the CDI based on the electrical conductivity measured on the cylindrical sample of the diameter of 1 cm and length of 1 cm, and the PEF batch system generated an exponential decay pulses of 143 µs width and electric field strength of 1.0 kV/cm. In the current study, monopolar rectangular pulses of electric field strength and pulse duration equaled, respectively, 1.07 kV/cm and 7 µs, were applied on the rectangular pieces of approximately 2 × 3 cm, with a thickness of around 5 mm, which affected the obtained values.

Generally, a higher energy input during PEF treatment results in a higher CDI of tissue [8]. The same gradual increase in CDI was observed in the current study with rising PEF energy. The CDI curve exhibited three distinct regions: 1. a low-energy zone (≤1–2 kJ/kg) associated with minor and probably reversible membrane permeabilization; 2. an intermediate zone (≈3–6 kJ/kg) characterized by a sharp increase in CDI, indicating probably the onset of irreversible electroporation; and 3. a high-energy zone (≥8 kJ/kg) where CDI values approached saturation, suggesting extensive cellular disruption. Based on the CDI–energy relationship, five representative energy levels (1, 2, 4, 5, 10 kJ/kg) from each group were selected for detailed analyses.

#### 2.1.2. Color of Red Bell Pepper Treated with PEF

**Figure 2:**
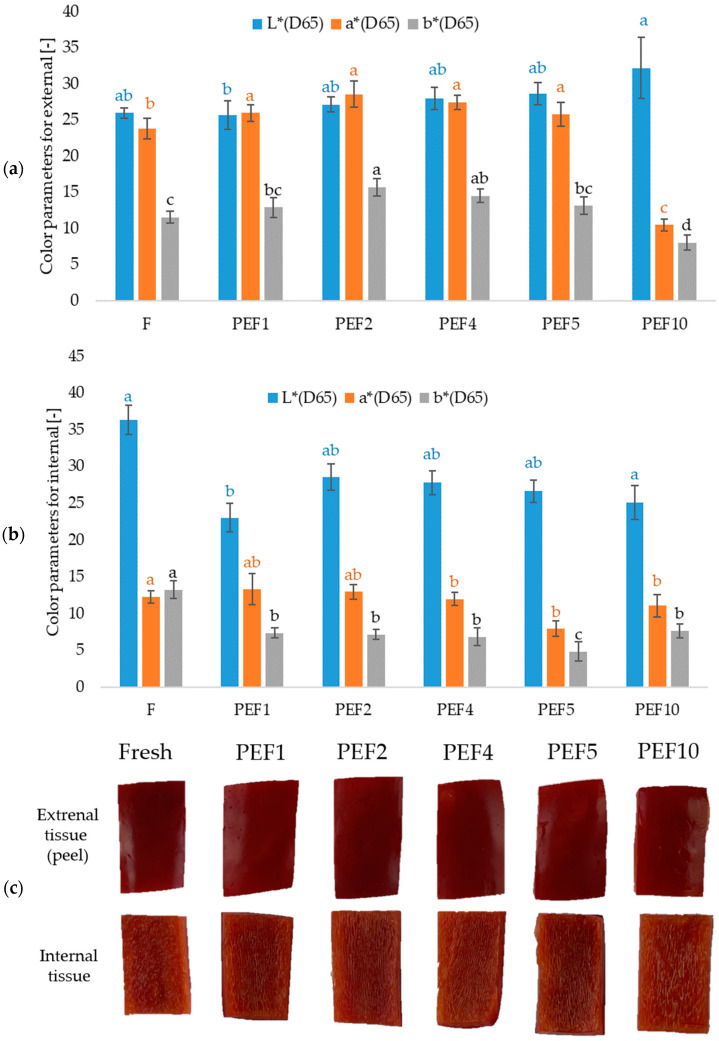
Color changes (L*, a*, b*) of (**a**) external tissue (peel) and (**b**) internal tissue of red bell pepper tissue after PEF treatment with applied different energies; (**c**) photographs of top and bottom of red bell pepper; a–d—different letters above the columns, marked with the same color, indicate different homogeneous groups for L* parameter, a* parameter, b* parameter, respectively (α = 0.05).

PEF treatment caused measurable alterations in the color attributes of the pepper skin (Figure 2). The fresh sample exhibited relatively low lightness (L* = 25.98) and high redness (a* = 23.79), characteristic of mature red bell pepper. Low- and medium-intensity PEF treatments (PEF1–PEF5) resulted in a moderate increase in L* (up to 28.68 for PEF5), accompanied by an increase in a* values (up to 28.54 for PEF2), indicating a visually redder color. These changes may reflect enhanced extraction or release of surface pigments due to partial electroporation. However, the most intensive treatment (PEF10) showed a markedly different color profile, with a substantial increase in L* (32.20) and a strong decrease in a* (10.47) and b* (8.05), indicating noticeable lightening and loss of red–yellow color. This suggests that high PEF intensity may induce pigment destabilization or structural changes that alter light scattering properties, resulting in a visibly paler and less saturated surface [15].

In contrast to the skin of the red bell pepper, the internal tissue exhibited a notable decrease in lightness (L*) following PEF treatment. The fresh sample showed the highest L* (36.36), while all treated samples had lower values (23.04–28.54), indicating pronounced darkening. The a* values remained stable across treatments. Whereas a consistent and substantial reduction in b* values (from 13.27 in fresh to 4.83–7.65 in treated samples) indicates reduced yellow color, likely due to cell membrane breakdown leading to pigment dilution, internal fluid redistribution, or initial stages of pigment oxidation.

The total color difference (ΔE) reported for the skin (external tissue) and internal tissue of red bell pepper after PEF treatment reveals distinct responses of the two tissues. For the skin, ΔE values ranged from 3.25 ± 1.25 (PEF1) up to 15.6 ± 1.84 (PEF10), which implies little visible change at low to moderate treatment intensities and a clearly noticeable color deviation at the highest PEF condition (Table 1). On the other hand, the internal tissue exhibited higher and more variable ΔE values (14.8 ± 1.69 at PEF1, decreasing to 7.6 ± 2.79 at PEF10), signifying that the inner parenchyma is more susceptible to structural and pigment-related change under PEF. Observed ΔE data indicate that for the skin of red bell pepper, moderate PEF intensities result in color change within the visually marginal range (ΔE < 5), which, according to criteria, may be considered noticeable but acceptable. In contrast, in the internal tissue of the red bell pepper, higher changes were observed; even moderate PEF intensities produce ΔE values exceeding 10, which approach levels observed in more aggressive processing in the literature, implying potentially perceptible quality loss. These results highlight that when applying PEF to whole fruit or minimally processed pepper, optimization must ensure that internal tissue color changes remain within acceptable thresholds, not just surface appearance.

The changes in the color of the external and internal tissue of the red bell pepper are presented in Figure 2c. Electroporation and partial loosening of epidermal cells can enhance pigment visibility and modify light scattering without immediately degrading carotenoids. Similar behavior has been observed when red bell peppers are pretreated with PEF and then dried by Won et al. [16]. They reported that PEF-pretreated red pepper retained higher a* and chroma values and exhibited lower overall color difference (ΔE*) during drying compared with untreated samples, which was attributed to improved carotenoid retention. The color changes observed in red bell pepper subjected to PEF treatment align well with mechanisms reported in the literature. Low and moderate field intensities (PEF1–PEF5) resulted in increased L* and a* values in the skin, indicating an enhancement of red color saturation. In contrast, the highest PEF intensity (PEF10) in the present study led to marked lightening of the skin (increased L*) combined with substantial reductions in a* and b*, reflecting pigment destabilization and loss of chromaticity. These processes are in line with the microstructural findings of Ade-Omowaye et al. [17], who showed that the degree of irreversible permeabilization in red bell pepper cells increases substantially with field strength and pulse number, altering mass transfer and optical properties. The internal tissue exhibited a distinctly different response, displaying considerable darkening (decreased L*) and a pronounced reduction in b*, indicating loss of yellow pigmentation. This pattern suggests greater susceptibility of parenchymal tissue to pigment dilution, internal water redistribution, and early oxidative reactions following extensive membrane breakdown. Comparable observations have been highlighted by Assaf et al. [18], who reported that PEF-induced structural weakening in the subcutaneous tissues of pepper can progress more rapidly than in the outer epidermal layers. The higher and more variable ΔE values recorded in the internal tissue (≥10 even at mild intensities) corroborate the literature describing the heightened vulnerability of internal plant tissues to oxidative and structural deterioration during strong electroporation events [17,19].

#### 2.1.3. Texture of Red Bell Pepper Treated with PEF

The texture profile analysis (TPA) revealed a clear reduction in hardness (Table 1) of red bell pepper tissue following PEF treatment compared with the untreated fresh sample. The fresh sample exhibited a hardness of 52.27 N, while all PEF-treated samples showed significantly lower values, ranging from 38.27 N (PEF4) to 43.25 N (PEF2). However, no statistically significant differences were observed among the various PEF treatment conditions themselves, indicating that although the process induced measurable softening of the tissue, increasing PEF intensity did not further intensify this effect from a statistical point of view. Still, there was a trend of decreasing tissue hardness with increasing PEF energy applied. Although SEM and micro-CT images revealed evident progressive structural damage with increasing PEF energy input, hardness values among PEF-treated samples did not differ significantly in several cases. This apparent discrepancy can be explained by the heterogeneity of tissue disruption, where localized microstructural collapse does not necessarily translate into statistically significant changes in macroscopic mechanical resistance. Moreover, texture profile analysis is inherently less sensitive to subtle, early-stage microstructural alterations than high-resolution imaging techniques, and therefore may not fully capture delicate structural modifications observed at the cellular level.

Furthermore, Pearson correlation analysis (Table S1) between cell disintegration index (CDI) and hardness provides additional information on texture. This variable showed a very strong, negative, and statistically significant correlation with CDI (r = −0.911), indicating that greater cell structure breakdown was associated with decreased hardness in red bell pepper. These findings are consistent with published studies indicating that PEF can modify the structural integrity of plant tissues by affecting cell wall cohesion or membrane permeability. Ali et al. [20] reported that PEF treatments may reduce hardness depending on the anatomical structure of the plant material and its susceptibility to membrane electroporation. Similarly, Li et al. [21] demonstrated that PEF-processed fresh-cut apples exhibited slightly reduced firmness relative to controls, reflecting minor structural softening following pulsed electric field exposure. The magnitude of softening observed in the present study (approximately 9–14 N below the untreated sample) falls within the range reported for other fresh vegetables and fruits subjected to non-thermal PEF treatments, confirming that while PEF effectively supports microbial inactivation, it introduces only moderate textural changes that remain acceptable for high-quality minimally processed produce.

#### 2.1.4. Structure of Red Bell Pepper Treated with PEF

**Figure 3:**
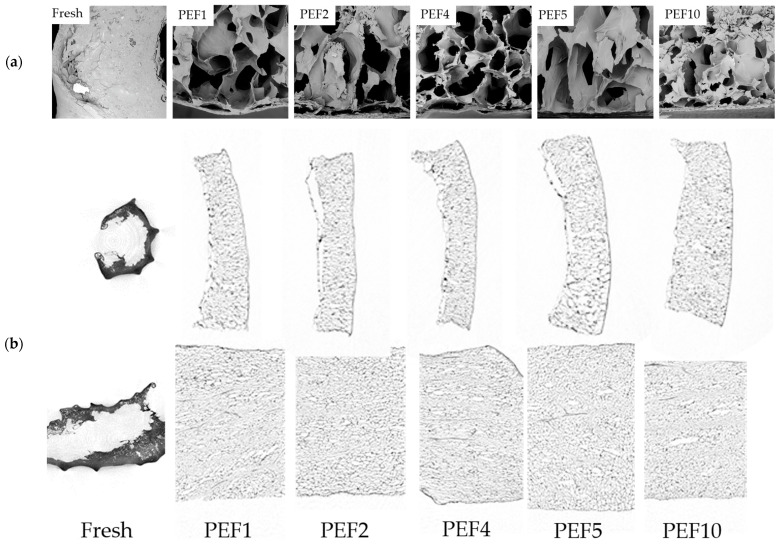
Effect of PEF treatment with applied different energies on structural changes in red bell pepper tissue (**a**) photographs from SEM (magnification 200×); (**b**) micro-CT visualization of whole sample and cross-section (raw images inverted in black and white colors to facilitate observation).

The structural alterations induced by PEF treatment were evaluated using scanning electron microscopy (SEM) and X-ray micro-computed tomography (micro-CT) (Figure 3). Both techniques consistently demonstrated progressive disruption of the red bell pepper matrix with increasing specific energy input, revealing distinct micro- and macrostructural changes.

In the fresh, untreated sample, SEM images show compact and continuous parenchyma. This dense structure corresponds to the high hardness values recorded for the control sample (Table 1), confirming the integrity of the cell membrane.

After PEF treatment, regardless of the energy used, the structure became looser, and significantly larger cell pores were observed in the tissue (Figure 3a,b). The pronounced structural differences between untreated and PEF-treated samples can be explained by the electroporation phenomenon induced by pulsed electric fields [10]. PEF treatment increases cell membrane permeability and loosens the structure of the cell wall–matrix network, resulting in partial tissue disintegration and a more porous, disrupted microstructure. Treatment at the lowest specific energy (PEF1, PEF2) reveals microstructural disruptions, whereas higher PEF levels result in more pronounced cell changes, including increased porosity and interconnected cells with intercellular spaces associated with tissue damage and the permeabilization process. In the cross-sectional micro-CT visualization, localized membrane perforation and slight enlargement of cells and intercellular spaces are revealed. This alteration is in line with the increase in CDI (Figure 1) and with the significant softening detected in texture measurements (Table 1). Similar effects of PEF on tissue porosity have been reported for apricot [22]. Also, increased pore formation reduces the mechanical strength, favoring subsequent processing. Furthermore, the formation of continuous pores and channels is clearly visible in PEF4–PEF10 samples (Figure 3b). Such connectivity is advantageous for industrial operations that rely on improved permeability, such as extraction or drying, as it reduces internal mass-transfer resistance [23]. Furthermore, this increased pore connectivity corresponds with the enhanced release of carotenoids and vitamin C, which are primarily located in chromoplasts and the cytosol. Conversely, the decrease in TPC and TFC suggests that phenolic compounds are susceptible to oxidation following permeabilization [24].

### 2.2. Effect of PEF Treatment on the Chemical Properties of the Red Bell Pepper

#### 2.2.1. Total Polyphenols Content (TPC), Total Flavonoids Content (TFC), Vitamin C (DHA + AsA) Content, and Total Carotenoid Content (TCC) of Red Bell Pepper Treated with PEF

One of the tasks of pulsed electric field (PEF) technology is to loosen the structure of the treated tissue in order to facilitate and improve diffusion-based processes, e.g., drying, freezing, or extraction [25,26,27]. This is due to the electroporation occurring during this process, which may be irreversible—leading to permanent cell damage (increased cell membrane permeability), or reversible—aimed, for example, at triggering a response of the treated tissue to a stress factor [28,29]. Table 2 presents the results of the determination of bioactive compounds such as total polyphenols content (TPC), total flavonoids content (TFC), vitamin C (DHA + AsA) content, and total carotenoid content (TCC) in unprocessed fresh red bell pepper (F) and matrices of the same material treated with PEF (various specific energy inputs, 1–10 kJ/kg).

The total polyphenol content (TPC) in fresh red bell pepper (F) was the highest (4152 ± 210 mg chlorogenic acid/100 g d.m.), whereas the application of PEF resulted in a decrease in TPC, the extent of which depended on the treatment energy. At the lowest levels of pulsed electric field energy (1–2 kJ/kg), the decrease was small and statistically insignificant compared to the control. At intermediate energy levels (4–5 kJ/kg), TPC decreased significantly, and the maximum effect was observed after PEF treatment at 10 kJ/kg (3617 ± 37 mg/100 g d.m.), representing a reduction of nearly 13% compared to the fresh sample. These changes indicate the existence of an intensity threshold, beyond which processes leading to the loss of polyphenolic compounds become dominant.

The obtained results are consistent with the electroporation effect: shorter and milder pulses increase membrane permeability and may facilitate the diffusion of compounds from vacuoles without their immediate degradation, whereas more intense fields cause more extensive damage to cellular structures, promoting contact between phenols and oxidoreductases as well as oxygen, and consequently leading to their oxidation [22,24]. Additionally, the increased permeability of the microstructure at higher energy levels may accelerate the release of phenols into the surrounding medium, where they undergo non-enzymatic transformations and/or bind with other matrix components, thereby reducing the measurable TPC, despite the initial increase in phenol release from the cells [30,31,32].

PEF pretreatment has been reported to enhance the retention and/or apparent extractability of phenolic compounds in freeze-dried fruit matrices; for instance, a significant increase in total phenolic content was observed in freeze-dried apricots after PEF application compared with non-treated controls [22]. In mushrooms, the impact of PEF on total phenolic content appears to be strongly dependent on the subsequent drying method and process conditions, as shown for button mushroom slices dried by hot air or electrohydrodynamic drying, where quality outcomes varied across pretreatments and drying regimes [33]. On the other hand, in the study by Baltacioglu et al. [34], it was found that in the case of purple-fleshed potatoes, the application of PEF treatment prior to hot-air and microwave drying resulted in a significant reduction in TPC in the tissue compared to the fresh material, whereas in yellow-fleshed potatoes, these changes were not statistically significant.

The total flavonoid content (TFC) in fresh red bell pepper (F) was 452 ± 28 mg QE/100 g d.m. and was significantly the highest among all samples (Table 2). The application of a pulsed electric field led to a decrease in TFC, with the magnitude of the decrease depending on the PEF energy. Even low-energy treatment (1 kJ/kg) caused a significant reduction in TFC, and further increasing the energy to 2 kJ/kg lowered the level to 326 ± 7 mg QE/100 g d.m. The lowest value was observed at 4 kJ/kg (292 ± 6 mg QE/100 g d.m.). At 5 kJ/kg, an increase in this parameter to 352 ± 12 mg QE/100 g d.m. was noted, whereas the highest tested energy (10 kJ/kg) again resulted in a reduction to 318 ± 4 mg QE/100 g d.m. This indicates a nonlinear response of the investigated plant matrix to the action of the pulsed electric field. The observed pattern of changes may be linked to the nature of PEF interactions with red bell pepper tissue. At low and moderate energy doses, increased membrane permeability promotes the release of compounds, but at the same time facilitates contact between flavonoids and oxidative enzymes as well as reactive oxygen species, leading to their degradation [35]. Further increases in energy, however, may deepen structural damage and intensify non-enzymatic oxidation, leading to a further decrease in TFC.

Additional information on the behavior of phenolic compounds is provided by the Pearson correlation analysis (Table S1) between the degree of cell disintegration (CDI) and the total polyphenol content (TPC) and flavonoid content (TFC). Both variables showed a very strong, negative, and statistically significant correlation with CDI (TPC: r = −0.980; TFC: r = −0.856), indicating that greater cellular structure disintegration was associated with lower retention of these compounds. This relationship suggests that under conditions of more severe cellular integrity disruption, polyphenols and flavonoids become more susceptible to leaching and undergo faster transformations detrimental to their stability. A similar relationship was reported by Carpentieri et al. [36], who demonstrated that an increase in the degree of cell disintegration (CDI) in grape pomace after PEF was directly linked to changes in flavonoid content, with their levels either increasing or decreasing depending on the intensity of tissue porosity induced by the pulsed electric field. In the study by Enjie et al. [37] on Bird’s Eye Chili (*Capsicum frutescens* L.), the use of PEF with an intensity of 3.1 kV/cm increased the total flavonoid content by 50.8% compared to samples not exposed to pulsed electric field, which the authors attributed to PEF-induced electroporation of cell membranes and the breakdown of intercellular pectin matrices.

In the case of vitamin C content, most of the samples contained the same amount of this chemical compound, taking into account the statistical point of view. Only the PEF1 sample exhibited significantly more vitamin C than the PEF4 sample (by 16%), and at the same time, it was the highest value (2325 ± 158 mg/100 g d.m.). In general, despite the lack of significant differences, the vast majority of samples treated with an electric field exhibited a slightly higher vitamin C content than the fresh sample. One of the goals of using pulsed electric fields is to loosen the treated tissue. This occurs through electroporation, which involves the generation of pores in the material’s cell membrane. This may result in increased permeability of the cell membrane of PEF-treated materials, allowing the release of certain substances from the disrupted tissue, thus increasing their extractability [38]. The beneficial effect of non-thermal PEF technology on the extraction of various chemical compounds has already been demonstrated, as evidenced by the example of microalgae—carotenoids [27], onion—phenolics [39], broccoli—sulforaphane [40], and nettle leaves—soluble proteins [41]. Considering that red bell pepper treated with PEF1 was characterized by the highest content of vitamin C and was the least processed with an electric field of all PEF-treated samples. This can be assumed that the cell disintegration index obtained by PEF1 (0.24 ± 0.01, Figure 1) may be the most optimal for the extraction of this substance from red bell pepper. Additionally, considering the lack of association between higher values of cell disintegration index and significant impact on vitamin C content, it is worth considering a less intense PEF treatment in the future as a sufficient procedure to support the extraction of this substance from the treated matrix, reducing the impact on environmental and energy issues.

Red bell peppers are vegetables that contain exceptionally high levels of naturally occurring beta-carotene [42]. The content of this pigment was expressed in this study as TCC (mg β-carotene/100 g d.m. (Table 2). Similarly to the determination of vitamin C content, in the case of TCC, it was possible to observe a higher beta-carotene content in red bell peppers treated with PEF than in the untreated ones. Nevertheless, no significant differences in TCC values were found between samples F, PEF1, PEF2, and PEF10. However, a significant increase in beta-carotene retention was observed in the tissue after medium-intensity PEF treatment (PEF4 and PEF5). Obtained results may indicate that the morphological changes caused by disruption of the red bell pepper tissue continuity by mild PEF treatments (1–2 kJ/kg), at a CDI level of 0.24–0.39, did not contribute to a noticeable release of beta-carotene from the structures of this matrix. In turn, intensive PEF processing (10 kJ/kg, CDI approximately 0.63) may have caused excessive tissue processing, resulting in beta-carotene leakage not only from cellular structures into the cell sap but also into the medium during PEF treatment (water) [28].

#### 2.2.2. Antioxidant Activity (ABTS, DPPH, FRAP) of Red Bell Pepper Treated with PEF

Antioxidants play a crucial role in maintaining human health, as they can limit or slow down adverse oxidative reactions [43]. Antioxidant activity is related to the chemical composition, primarily due to the presence of polyphenolic compounds and other biologically active substances [44]. In evaluating antioxidant properties, the most commonly used methods are the ABTS and DPPH assays, which are based on the ability of antioxidant compounds to reduce the respective DPPH and ABTS radicals to their stable, non-radical forms. Another widely used analytical tool is the FRAP assay, which determines reducing potential, a key mechanism of antioxidant action [45]. For this reason, three tests (ABTS, DPPH, and FRAP) were used in this study to determine the antioxidant activity of the samples.

In red bell pepper samples subjected to PEF, antioxidant activity (ABTS, DPPH, FRAP) changed nonlinearly with treatment energy, as shown in Table 3. The strongest decline was observed for DPPH: from 29.4 mg TE/g d.m. (F) to approximately 19–22 mg TE/g d.m. across all PEF-treated variants (PEF1–PEF10). FRAP decreased at every pulsed electric field energy dose, particularly from 43.0 ± 1.52 (F) to 25.1 ± 0.76 mg TE/g d.m. (PEF10), while at moderate energies (4–5 kJ/kg) it remained at 31–32 mg TE/g d.m. ABTS remained relatively stable (14.3–17.7 mg TE/g d.m.), with a slight reduction at 1–2 kJ/kg and leveling off at 4–10 kJ/kg. The data indicate that PEF primarily reduces electron-transfer capacity (DPPH/FRAP), with a smaller effect on ABTS cation-radical scavenging.

The correlation matrix confirms these relationships (Table S1). DPPH and FRAP were strongly correlated (r = 0.871), and DPPH showed a strong positive correlation with TFC (r = 0.901), whereas ABTS exhibited weak and non-significant relationships with TPC/TFC. TPC correlated with TFC (r = 0.850), and its relationships with DPPH/FRAP were close to significance (r ≈ 0.80; *p* ≈ 0.05–0.07). This indicates that, in the studied system, antioxidant capacities measured by DPPH/FRAP were mainly determined by flavonoids and polyphenols, whereas the ABTS response was independent of these fractions.

In red bell pepper samples, the use of PEF resulted in a pronounced decrease in activity, as measured by DPPH and FRAP, particularly at 10 kJ/kg, while ABTS values remained relatively stable. The decline in reducing capacity in the DPPH/FRAP assays can be interpreted as a reduction in the pool of compounds capable of donating electrons (mainly phenols and flavonoids), consistent with the characteristics of these methods [46].

From a process perspective, this pattern aligns well with the mechanism of PEF action. Electrical pulses cause electroporation of membranes, forming pores that increase cell permeability, facilitating water and solute diffusion, and accelerating mass transfer. Consequently, released phenols and carotenoids come into easier contact with oxygen [13]. Segovia et al. [47] reported an increase in oxygen radical absorption capacity from 2% to nearly 14% in *Borago officinalis* L. leaves due to PEF treatment (300 Hz, 30 kV). In studies by Huang et al. [48], PEF treatment preserved the most β-carotene and increased the antioxidant activity of dried apricots. Conversely, Quitão-Teixeira et al. [49] found no statistically significant differences in antioxidant activity between the control sample and carrot juice treated with pulsed electric field.

The obtained results suggest that for red bell pepper, lower PEF energies (1–4 kJ/kg) are beneficial, as they limit the decrease in FRAP and DPPH while not substantially reducing ABTS results. Exceeding 10 kJ/kg in the experiment was associated with a clear decline in reducing capacity, consistent with reports that intense PEF can increase phenol oxidase activity and accelerate browning and phenol degradation [50].

#### 2.2.3. Sugar Composition of Red Bell Pepper Treated with PEF

The results regarding the content of three sugars (sucrose, glucose, and fructose) in all red bell peppers (untreated and PEF-treated with specific energy inputs in the range of 1–10 kJ/kg) are displayed in Table 4. Interestingly, the content of determined sucrose decreased, with a gradual increase in specific energy input, and the maximum level was reached in samples PEF1 and PEF2. After such mild PEF treatment, the materials were characterized by a 48–62% higher content of this disaccharide than the fresh sample (0.73 ± 0.15 g/100 g d.m.), which were the highest recorded values among all samples. This could be related to the opening of cellular structures within the treated tissue, allowing for increased transport of this chemical compound into the cell sap [51]. The lowest sucrose content was found in the PEF10 sample, which indicates that significant disruption of tissue continuity (CDI = 0.63 ± 0.01, Figure 1) may cause leakage of some substances into the medium in which the sample is immersed during processing [52]. Mild PEF treatment induced similar trends for glucose. The highest content of this monosaccharide was observed in samples PEF1 and PEF2, while the lowest was determined in sample PEF5. In turn, as for the second monosaccharide, fructose, its significantly lower content compared to the other red bell peppers was recorded in the most PEF-processed samples (PEF5 and PEF10). The obtained results demonstrate that a smaller scale of tissue damage caused by PEF may be more beneficial from the perspective of extracting selected compounds from cellular structures, while limiting the risk of their leakage into water during the PEF procedure.

#### 2.2.4. Fourier-Transform Infrared Spectroscopy (FTIR) of Red Bell Pepper Treated with PEF

**Figure 4:**
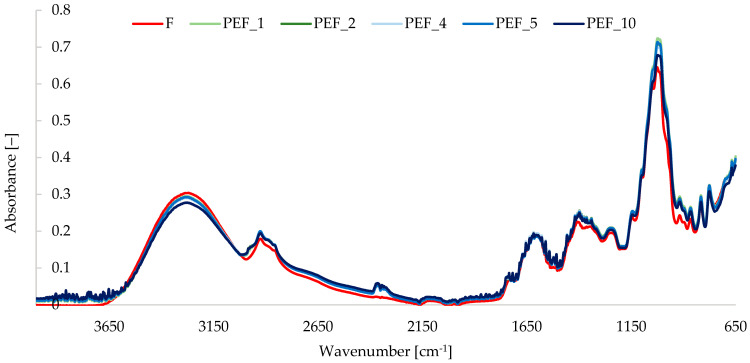
FTIR spectra of red bell pepper tissue after PEF treatment with applied different energies.

PEF treatment did not alter the overall FTIR band pattern of red bell pepper tissue (Figure 4), indicating no formation of new functional groups; however, it clearly affected band intensities in an energy-dependent manner.

All samples showed a broad O–H stretching band at 3600–3000 cm^−1^, C–H stretching around 2925 and 2855 cm^−1^, a carbonyl band near 1740 cm^−1^, and complex carbohydrate/pectin vibrations in the 1200–900 cm^−1^ region [53,54]. Compared with fresh tissue, the FTIR spectra revealed a gradual decrease in the relative intensity of the O–H band with increasing specific energy, most pronounced at 10 kJ/kg, suggesting reorganization of the hydrogen-bond network and partial dehydration of the cell-wall matrix. In contrast, bands assigned to C–H stretching and to C–O/C–O–C vibrations of polysaccharides (especially 1150–950 cm^−1^) increased for PEF 1–5 kJ/kg and slightly leveled off at 10 kJ/kg, consistent with enhanced exposure or extractability of cell-wall carbohydrates after electroporation. Comparable PEF-induced changes in the O–H and carbohydrate regions were reported for soluble dietary fiber from peanut shells [55], cereal polysaccharide fractions [56], and inulin obtained from *Agave americana* [57], where FTIR primarily reflected structural loosening and increased polysaccharide accessibility. The present data for red bell pepper is in line with this interpretation and indicate that moderate energy inputs (1–5 kJ/kg) primarily rearrange water–polysaccharide interactions, whereas the highest energy (10 kJ/kg) is associated with more advanced dehydration and partial collapse of the hydrogen-bonded network, as evidenced by the attenuated O–H band.

#### 2.2.5. Thermogravimetric Analysis (TGA) of Red Bell Pepper Treated with PEF

According to the thermogravimetric data (Table 5), red bell pepper samples exhibited a four-step degradation profile characteristic of carbohydrate-rich plant matrices. The first mass-loss step (30–110 °C), reflecting moisture evaporation, showed similar values across treatments (3.9–4.8%). However, the decomposition temperature decreased from 77 °C in the fresh sample to 63–68 °C after PEF, indicating the presence of more weakly bound water in electroporated tissues. The second stage (110–250 °C), associated with degradation of sugars and hemicellulosic components, was slightly more pronounced in PEF samples (34.4–35.1%) compared with the untreated tissue (32.1%). In contrast, the major decomposition step (250–480 °C), representing the breakdown of cellulose-dominated polysaccharides, remained highly stable, with Tmax values consistently at 310–311 °C, regardless of the PEF energy. The final stage (480–600 °C) contributed only marginally (2.3–2.5%), reflecting oxidation of the carbonaceous residue. The nearly unchanged Tmax values across all stages confirm that PEF treatment did not alter the intrinsic thermal stability of key biopolymer fractions.

Similar behavior has been reported for other PEF-treated plant systems. Faridnia et al. [58] showed that, although PEF (1–10 kJ/kg) substantially modified the microstructure and permeability of whole potato tubers, the solid matrix remained structurally intact, indicating preserved stability of cell-wall polymers. Parniakov et al. [59] observed enhanced extraction of bioactive compounds from mango peels after PEF or high-voltage electrical discharge, without evidence of extensive thermal degradation of the polysaccharide backbone. Moreover, Dellarosa et al. [60] demonstrated that PEF-induced electroporation in mushroom stalks primarily affected water distribution and loss, while the solid tissue framework remained conserved. Taken together, these studies support the conclusion that, within the applied energy range, PEF reorganizes water binding and microstructure in red bell pepper but does not compromise the intrinsic thermal resilience of its polysaccharide matrix.

#### 2.2.6. Time Domain Nuclear Magnetic Resonance (TD-NMR) of Red Bell Pepper Treated with PEF

**Figure 5:**
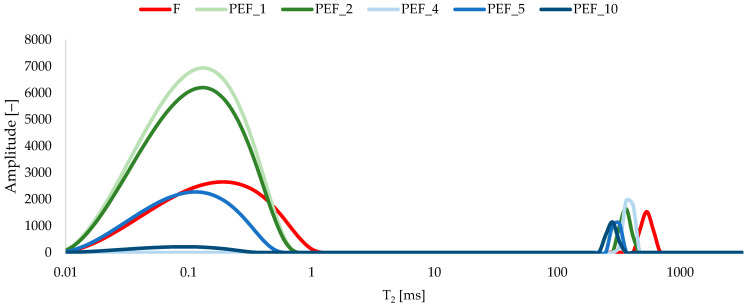
T_2_ spectra (examples) of red bell pepper tissue after PEF treatment with applied different energies.

The TD-NMR relaxation time distributions of red bell pepper clearly indicate the presence of several water populations differing in mobility and degree of binding within the tissue microstructure (Figure 5).

In the fresh sample, the signal is dominated by a long-T_2_ component, attributable to vacuolar and intercellular water, accompanied by one or more shorter-T_2_ components corresponding to water associated with cell walls, pectic polysaccharides, and other macromolecular structures. After PEF treatment, the relative contributions of these components change in an energy-dependent manner, reflecting progressive loss of membrane integrity and reorganization of the aqueous compartments. At lower specific energies, the increase in the shorter-T_2_ fraction together with a partial reduction in the long-T_2_ signal suggests a temporary increase in water–matrix interactions and partial immobilization of water in the electroporated tissue. At higher energies, the predominance of longer relaxation times and the attenuation of short-T_2_ contributions are consistent with more extensive loss of cellular compartmentalization and the formation of larger, less restricted water domains in disrupted parenchyma. These trends align with previous TD-NMR studies on PEF-treated plant tissues. Dellarosa et al. [61] showed that PEF of apple tissue caused a redistribution of water between vacuole, cytoplasm, and extracellular space, with increased longest T_2_ and diffusion coefficient as membrane disruption intensified. Tylewicz et al. [62] reported that PEF treatment of apple followed by freeze-drying led to clear changes in TD-NMR water populations, indicative of altered integrity and continuity of the cell structure. Ersus et al. [63] demonstrated for onion tissue that changes in specific T_2_ components measured by ^1^H-NMR relaxometry closely reflected the degree of electroporation-induced cell damage. Available literature data indicate that, in red bell pepper, PEF primarily modulates water distribution and mobility through structural disruption, while preserving the overall pattern of water populations typical for parenchymatous tissue [19,64].

### 2.3. Effect of PEF Treatment on Microbial Stability of the Red Bell Pepper

**Figure 6:**
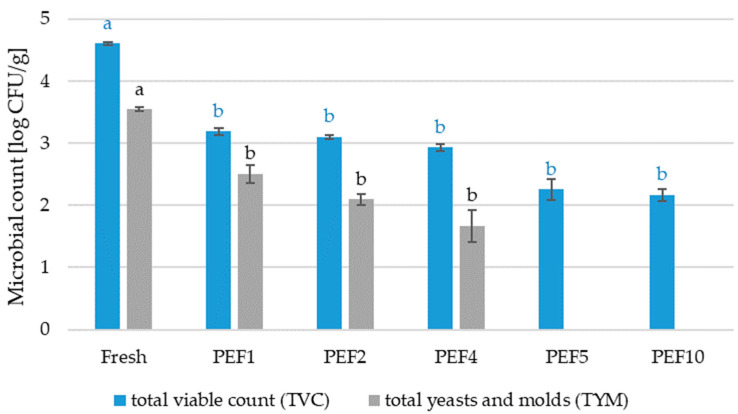
Microbial quality (total viable count (TVC) and total yeasts and molds (TYM)) of red bell pepper tissue after PEF treatment with applied different energies; a, b—different letters above the columns, marked with the same color, indicate different homogeneous groups for TCV and TYM, respectively (α = 0.05).

The application of PEF resulted in a clear, intensity-dependent reduction in both total viable count (TVC) and yeasts and molds (TYM) compared to the fresh red bell pepper (Figure 6). The initial TVC of 4.60 log CFU/g in untreated red bell pepper decreased to 3.19–3.10 log CFU/g for PEF1–PEF2 and further to 2.93 log CFU/g at PEF4, with the most intensive treatments (PEF5 and PEF10) yielding 2.25 and 2.17 log CFU/g, respectively, e.g., a maximum reduction of ~2.4 log. For TYM, the fresh sample contained 3.54 log CFU/g, which was reduced to 2.50, 2.09, and 1.66 log CFU/g after PEF1, PEF2, and PEF4, and fell below the detection limit (1 log CFU/g) after PEF5 and PEF10, indicating complete inactivation or suppression of detectable fungal growth at higher field intensities. Statistical analysis showed the effect of pre-treatment on the reduction in the total count of microorganisms regardless of the PEF intensity used, which indicates that even a small dose of PEF can extend the shelf life of red bell peppers.

These results are consistent with the known mechanism of PEF, where short high-voltage pulses induce electroporation and loss of integrity of microbial cell membranes, leading to log-scale reductions in vegetative cells while preserving product quality. Comprehensive reviews report typical reductions of 1–3 log CFU in complex food matrices, especially solid fruits and vegetables, with efficacy strongly influenced by matrix conductivity and structural heterogeneity. The maximum TVC reduction observed here (~2.4 log) is therefore within the expected range for fresh plant tissue, while the complete loss of detectable TYM at higher PEF levels is particularly notable given that yeasts and molds are often reported to be more resistant than bacteria, as shown for *Candida albicans* in sugarcane juice, where fungal cells survived conditions that fully inactivated *Escherichia coli* [24,65].

### 2.4. Effect of PEF Treatment on Red Bell Pepper Tissue

**Figure 7:**
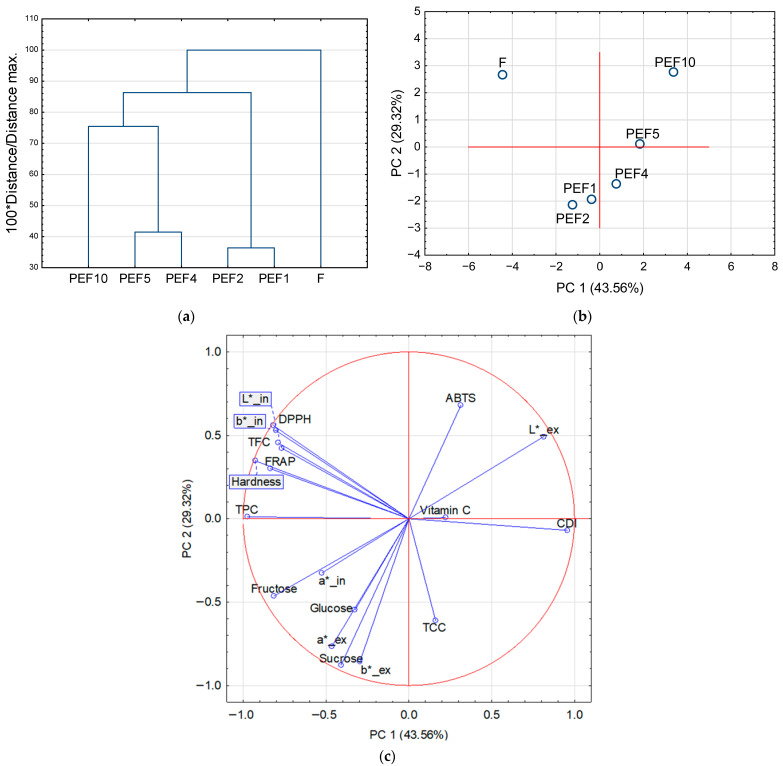
(**a**) Hierarchical cluster analysis (HCA, Ward’s method, Euclidean distance) of red bell pepper samples treated with increasing PEF energy; (**b**) Principal Component Analysis (PCA)—score plot showing the distribution of samples; (**c**) Principal Component Analysis (PCA)—loading plot illustrating the relationships among the analyzed variables.

The dendrogram presents four clearly separated clusters, indicating differentiated responses of the pepper tissue to increasing PEF energy input (Figure 7a). The first cluster consisted solely of the Fresh sample (control), representing structurally intact tissue. The second cluster grouped PEF1 and PEF2 samples, corresponding to mild and largely reversible electroporation. The third cluster, composed of PEF4 and PEF5, reflected the intermediate-energy range where irreversible electroporation dominated. Samples in this cluster showed increased CDI. Finally, the fourth cluster, represented by PEF10, was clearly distinct from all others, indicating the most intense structural changes, such as high porosity and extensive cell wall disruption, as well as the highest changes in color and texture properties.

Principal component analysis (PCA) was applied to visualize multivariate differences among samples subjected to increasing PEF energy levels, based on the following variables: CDI, TPC, TFC, TCC, vitamin C, ABTS, DPPH, FRAP, sucrose, glucose, fructose, hardness, and color parameters of the external (L*_ex, a*_ex, b*_ex) and internal tissues (L*_in, a*_in, b*_in). The PCA score plot supported the clustering pattern observed in the HCA (Figure 7b). The first two principal components explained 43.56% (PC1) and 29.32% (PC2) of the total variance, together accounting for 72.88% of the overall data variability.

Variables related to structural integrity, including hardness, were located on the negative side of PC1, together with total phenolic content (TPC), total flavonoid content (TFC), and antioxidant activity parameters (DPPH and FRAP). The similar orientation of these vectors indicates strong positive correlations among these variables. In contrast, the cell disintegration index (CDI) was positioned on the positive side of PC1, showing a strong negative association with hardness and phenolic-related parameters, reflecting progressive tissue disruption induced by increasing PEF energy input. Antioxidant activity assessed by ABTS was mainly associated with positive PC2 values, indicating a partially distinct behavior compared with DPPH and FRAP assays. Color parameters of the external tissue (L*_ex) showed positive loadings on both PC1 and PC2, whereas internal tissue color coordinates (L*_in and b*_in) were located in the upper-left quadrant, indicating their association with higher phenolic content and tissue firmness. Sugar-related variables (sucrose, glucose, and fructose) clustered predominantly in the lower-left quadrant, showing negative loadings on both PC1 and PC2, which suggests close interrelationships among soluble sugars and an inverse association with the degree of tissue disintegration. Total carotenoid content (TCC) was mainly associated with negative PC2 values and was located close to vitamin C, which exhibited a moderate contribution to PC1 and a relatively weak contribution to PC2.

The combined dendrogram and PCA results demonstrate an energy-dependent transformation of pepper tissue across four well-defined stages: (1) intact tissue (F), (2) mildly permeabilized (PEF1, PEF2), (3) irreversibly electroporated (PEF4, PEF5), and (4) severely disintegrated (PEF10). This progression integrates structural, mechanical, and compositional changes and indicates that moderate PEF levels (4–5 kJ/kg) represent an optimal range that balances the enhanced release of carotenoids and vitamin C with acceptable texture preservation. However, this range was associated with a decrease in the TPC and TFC as well as antioxidant activity. The clear separation of PEF10 suggests that excessive energy input leads to over-disintegration and potential quality deterioration [28].

## 3. Materials and Methods

### 3.1. Materials

Red bell peppers (*Capsicum annuum* L.), cv. ‘California Wonder’, were obtained from a local wholesale market in Bronisze (Poland). After delivery, the fruit was stored under refrigerated conditions (4–5 °C, 85–95% relative humidity) and all experiments were carried out within one week of purchase. Only sound peppers of comparable size and firmness, with ripeness assessed visually based on uniform red coloration, and free from visible defects or mechanical damage, were selected for the study. Before processing, the peppers were washed with cold water, gently dried with paper towels, and deseeded. The pericarp was then cut into rectangular pieces of approximately 2 × 3 cm, with the slice thickness adjusted to about 5 mm. Each PEF treatment was performed on three independent batches of red bell pepper. All physicochemical and microbiological analyses were conducted in triplicate (n = 3). Color measurements were carried out in six replicates per sample (n = 6), whereas texture measurements were performed in ten replicates per sample (n = 10). The results are presented as mean ± standard deviation.

### 3.2. Sample Preparation (PEF Treatment)

Red bell pepper tissue was pretreated with pulsed electric fields (PEF) in a laboratory-scale batch system (PEFPilot™ Dual System, Elea Vertriebs- und Vermarktungsgesellschaft mbH, Quakenbrück, Germany) [66]. For each run, approximately 250 g of pepper pieces were placed between two parallel stainless-steel electrodes in a treatment chamber and covered with tap water to reach a total load of 1.0 kg (sample + medium). During PEF treatment, the samples were arranged parallel to the direction of the electric field flow. The process medium had a temperature of about 21 °C and an electrical conductivity of ~0.7 mS/cm. The temperature was also measured after the treatment; however, no significant changes were observed (21 ± 0.2 °C). PEF was applied using monopolar rectangular pulses at a fixed electric field strength of 1.07 kV/cm and a pulse duration of 7 µs. The electric field strength was fixed due to the operational characteristics of the Elea system and the applied treatment chamber configuration and could not be independently adjusted in this set-up. The PEF treatment was performed in a PEFPilot™ Dual batch chamber with two parallel stainless-steel electrodes and an electrode gap (d) of 24 cm, consistent with the standard Elea chamber configuration. The specific energy input (W_spec, kJ/kg) was adjusted in the range 0–10 kJ/kg by varying the number of pulses (0–1112) at a constant pulse frequency of 24 Hz. Voltage and pulse current were continuously monitored by the system to calculate and verify the applied specific energy input (W_spec). For a parallel-plate batch system, the specific energy input was calculated according to:

W_spec = (*U* × *I* × *n* × *t_p_)*/*m*, (1)

where *U* is the electrode voltage (V), *I* is the current intensity (A), *n* is the number of pulses (−), *tₚ* is the duration of a single pulse (s), and *m* is the total mass of the treatment chamber content (kg). The electric field strength (E, kV/cm) was related to the applied voltage and electrode gap (*d*, cm) by:

E = *U*/*d*, (2)

Based on Equation (2), the corresponding pulse voltage for 1.07 kV/cm and an electrode gap of 24 cm was approximately 25.7 kV.

Immediately after treatment, the pepper pieces were gently blotted with paper towels to remove surface moisture and directed to further analyses. All physicochemical measurements were completed within 15 min after PEF application to minimize time-dependent changes in tissue properties. All obtained samples and their codes are given in Table 6.

The extent of electroporation in red bell pepper tissue was quantified using the cell disintegration index (CDI), calculated from electrical conductivity measurements. For this purpose, samples were treated at specific energy inputs from 0 to 12 kJ/kg (0–9 kJ/kg with 0.5 kJ/kg increments and 10–12 kJ/kg with 1 kJ/kg increments), and the conductivity of the pepper tissue (σ) was recorded using a conductivity meter equipped with an immersion cell. Samples for the CDI curve were randomly selected from each treatment group. CDI values were calculated from triplicate measurements. disintegrated tissue (σ_d_) was obtained by subjecting pepper pieces to three successive freeze–thaw cycles (freezing at −18 °C for 24 h and subsequent thawing at 25 °C for 4 h), which ensured complete rupture of cell membranes. After the third cycle, the electrical conductivity reached a stable maximum, indicating full loss of membrane integrity. Intact tissue (σ_i)_ was represented by the untreated control. CDI was then computed according to Lebovka et al. [67] as:

CDI = (σ − σ_i_)/(σ_d_ − σ_i_), (3)

where σ, σ_d_, and σ_i_ are the conductivities of PEF-treated, totally disintegrated, and intact tissue, respectively. This approach, widely applied for characterizing PEF-induced damage in plant matrices, provides a dimensionless index ranging from 0 (no disintegration) to 1 (complete disintegration).

### 3.3. Physico-Chemical Characterization

#### 3.3.1. Color

**Figure 8:**
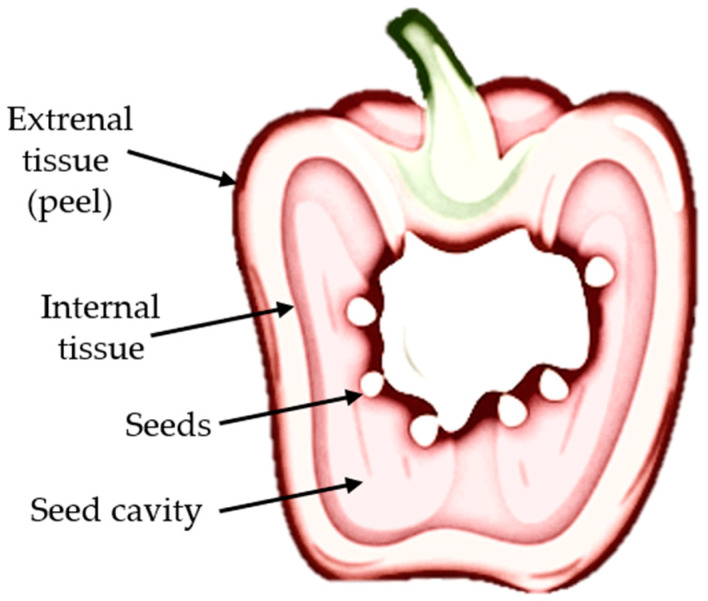
Cross-sectional schematic of red bell pepper illustrating the defined sampling regions: external tissue (peel), internal tissue (tissue located inside the seed cavity), and the seeds and seed cavity.

Color measurements of red bell pepper were performed in the CIE Lab* space using a CR-400 colorimeter (Konica Minolta, Osaka, Japan) with D65 illumination and an 8 mm aperture [68]. The instrument was calibrated in reflectance mode against a standard white tile and black trap supplied by the manufacturer. Pericarp color was recorded on both the external (peel) and internal surfaces (Figure 8); for each side, six readings were taken at randomly selected positions, and mean L*, a*, and b* values were calculated. Total color difference (ΔE) was determined relative to the non-treated control, with higher ΔE values indicating greater deviation from the reference.

Macroscopic appearance of the peppers was documented using a Nikon D7000 digital camera (Nikon, Tokyo, Japan) fitted with a 105 mm lens, positioned 50 cm from the sample. Illumination was provided by four 6500 K fluorescent daylight lamps arranged at 45° to the sample in a light-diffusing, shadow-free chamber to ensure uniform lighting and minimize reflections.

#### 3.3.2. Texture

Texture profile analysis (TPA) of red bell pepper tissue was carried out using a TA.XT2i texture analyzer (Stable Micro Systems, Surrey, UK) [69]. From each sample, cylindrical pieces of pericarp with a diameter of 5 mm were obtained using a cork borer, ensuring a uniform diameter and comparable height of all specimens. The cylinders were positioned centrally under the 20 mm Ø compression platen (P/20 probe). Measurements were conducted in compression (distance-controlled) mode with the following instrument settings: pre-test speed 1 mm/s, test speed 5 mm/s, post-test speed 1 mm/s, penetration depth 10 mm, and an automatic trigger force of 50 g. Each specimen underwent a double-compression test (two consecutive cycles) with a 5 s interval between the first and second compression. Hardness was used as the principal TPA parameter and was defined as the maximum force recorded during the first compression cycle. For each processing treatment, texture was assessed on ten individual specimens.

#### 3.3.3. Bioactive Compounds (Total Polyphenols Content, Total Flavonoids Content, Vitamin C (DHA + AsA) Content, Total Carotenoids Content) and Antioxidant Activity

##### Extraction for Total Phenolics, Flavonoids, and Antioxidant Assays (ABTS, DPPH, FRAP)

Prior to chemical analyses, red bell pepper tissue was ground in an analytical mill (A11 basic, IKA-Werke GmbH, Staufen, Germany). The material was extracted with 80% (*v*/*v*) ethanol. For this purpose, weighed portions of the sample were combined with the solvent and shaken for 12 h at 16 °C on a laboratory shaker (Multi Reax, Heidolph Instruments, Schwabach, Germany) [70]. After extraction, the suspensions were clarified by centrifugation at 4350 rpm for 4 min (MegaStar 600, VWR, Leuven, Belgium), and the clear supernatants were used for spectrophotometric determinations of TPC, TFC, and antioxidant activity.

The moisture content of the ground pepper tissue was determined using the vacuum-oven method [71]. Aliquots of the sample were dried in a vacuum oven (Memmert VO400, Schwabach, Germany) at 70 °C and 10 mPa for 24 h, and the dry matter content was calculated from the mass loss. The contents of bioactive compounds were expressed on a dry matter basis (per g or per 100 g d.m., as specified in each assay).

##### Total Polyphenols Content (TPC)

The total phenolic content was quantified using the Folin–Ciocalteu reagent [72]. An aliquot of the ethanolic extract (10 µL) was dispensed into a 96-well microplate and diluted with distilled water. Subsequently, 40 µL of Folin–Ciocalteu reagent (previously diluted 1:5 with water) was added. After a reaction time of 3 min, 250 µL of a 7% (*w*/*v*) Na_2_CO_3_ solution was added to each well. The mixtures were kept for 60 min at room temperature in the dark, and the absorbance was then recorded at 750 nm using a Multiskan Sky microplate reader (Thermo Electron, Waltham, MA, USA). Calibration was carried out with chlorogenic acid solutions (0–100 µg/mL), and the results were expressed as mg chlorogenic acid equivalents per 100 g dry matter (d.m.). All samples were analyzed in triplicate.

##### Total Flavonoids Content (TFC)

Total flavonoids were determined by an aluminum chloride–based colorimetric assay [73]. Portions of the ethanolic extract (20 µL) were pipetted into a 96-well microplate, followed by 80 µL of distilled water and 10 µL of 5% (*w*/*v*) NaNO_2_ solution. After 5 min, 10 µL of a 10% (*w*/*v*) AlCl_3_ solution was added. The reaction was allowed to proceed for 6 min, after which 40 µL of 1 M NaOH was added, and the volume was adjusted with distilled water to achieve a measurable absorbance. After 20 min of incubation at room temperature, the absorbance was read at 510 nm (distilled water used as a reference). Quantification was performed using a quercetin calibration curve (0–500 µg/mL), and the data were expressed as mg quercetin equivalents per 100 g d.m. Each sample was measured in triplicate.

##### Vitamin C (DHA + AsA) Content

Total vitamin C (ascorbic acid + dehydroascorbic acid) was determined by UPLC with photodiode array detection. The analysis was carried out on an ACQUITY UPLC H-Class system (Waters, Milford, MA, USA) equipped with a PDA detector, following the chromatographic conditions described previously [74]. To minimize oxidative losses, extraction was performed immediately after sampling and under limited light exposure. Portions of fresh pepper tissue (0.5 g) were weighed into pre-cooled tubes and homogenized with 10 mL of chilled extraction medium containing 3% (*w*/*v*) metaphosphoric acid and 8% (*v*/*v*) acetic acid. The homogenates were vortexed for 10 min and then centrifuged at 6000 rpm for 5 min at 4 °C.

To obtain total vitamin C, the resulting supernatant was mixed 1:1 (*v*/*v*) with dithiothreitol (DTT) solution (1 g L^−1^) and kept for 60 min at 4 °C to ensure complete reduction of dehydroascorbic acid to ascorbic acid. Prior to injection, the extracts were filtered through 0.22 µm PSF GHP syringe filters (Pall, Ann Arbor, MI, USA). Chromatographic separation was performed on an ACQUITY UPLC HSS T3 column (2.1 × 100 mm, 1.8 µm; Waters, Wexford, Ireland) with an injection volume of 5 µL. The column temperature was maintained at 25 °C, the autosampler at 4 °C, and the mobile phase flow rate was 0.25 mL/min. Detection was set at 245 nm. Quantification was based on an external calibration curve prepared from L-ascorbic acid standards (0.005–0.10 mg/mL). All samples were analyzed in triplicate.

##### Total Carotenoid Content (TCC)

Total carotenoids were determined spectrophotometrically according to Janiszewska et al. [75]. Ground pepper tissue (1.5 g) was mixed with 20 mL of water and 1 mL each of Carrez I and Carrez II solutions to precipitate proteins and polysaccharides. After centrifugation (5 min, 2000× *g*), the residue was extracted three times with 20 mL portions of acetone. The combined acetone extracts were transferred into a separating funnel and extracted with 40 mL of petroleum ether. Phase separation was facilitated by adding 10 mL of water. The carotenoid-rich ether phase was dried over anhydrous Na_2_SO_4_ (1.5 g) and then made up to 100 mL with petroleum ether. Absorbance was measured at 450 nm using a Spectronic 200 spectrophotometer (Thermo Fisher Scientific Inc., Waltham, MA, USA). TCC was calculated as β-carotene equivalents using an extinction coefficient of 2592 for petroleum ether. Measurements were performed in triplicate.

##### Antioxidant Activity (ABTS, DPPH, FRAP)

The extraction procedure used for antioxidant assays was performed as described in Section Extraction for Total Phenolics, Flavonoids, and Antioxidant Assays (ABTS, DPPH, FRAP). Antioxidant capacity was assessed by ABTS•^+^ and DPPH• radical scavenging assays, together with the FRAP method. Stock radical solutions were prepared 24 h prior to analysis. The DPPH• solution was obtained by dissolving 25 mg of 2,2-diphenyl-1-picrylhydrazyl (Sigma-Aldrich, St. Louis, MO, USA) in methanol. The ABTS•^+^ solution was prepared by dissolving 38.4 mg of ABTS (2,2′-azino-bis(3-ethylbenzothiazoline-6-sulfonic acid)) and 6.6 mg of potassium persulfate in 10 mL of distilled water and then allowing the mixture to stand in the dark at room temperature for 16–24 h to complete radical generation. Before measurement, both radical solutions were diluted with 80% (*v*/*v*) ethanol to achieve an absorbance of approximately 0.7 at 515 nm (DPPH•) or 734 nm (ABTS•^+^). All reactions were conducted in 96-well microplates by adding 10 µL of ethanolic pepper extract to 250 µL of the respective radical solution. For the DPPH• assay, plates were incubated in the dark for 30 min, and absorbance was recorded at 515 nm. For the ABTS•^+^ assay, absorbance was measured after 6 min at 734 nm. In both methods, 80% ethanol was used for the blank. Radical scavenging activity was calculated from the decrease in absorbance of the radical solutions in the presence of the extract and expressed as Trolox equivalents (mg Trolox/g d.m.), using Trolox calibration curves prepared in 80% ethanol [76,77].

Ferric reducing antioxidant power (FRAP) was evaluated according to Solaberrieta et al. [78] with freshly prepared reagents. The working FRAP solution was obtained by mixing 0.3 mol/L acetate buffer (pH 3.6), 10 mmol/L TPTZ in 40 mmol/L HCl, and 20 mmol/L FeCl_3_·6H_2_O at a ratio of 10:1:1, followed by preheating to 37 °C. In a microplate, 180 µL of the FRAP reagent was combined with 5 µL of sample extract, shaken gently, and incubated for 15 min at 37 °C in the dark. Absorbance was then measured at 593 nm. Trolox standards were analyzed in parallel, and FRAP values were expressed as µmol Trolox/g d.m. Each sample was assessed in triplicate.

#### 3.3.4. Sugar Composition

Individual sugars were quantified by HPLC equipped with a refractive index detector (Waters, Milford, MA, USA). Fresh pepper tissue (1.0 g) was weighed into screw-cap tubes and extracted with 10 mL of Milli-Q water (18.2 MΩ·cm) at 80 °C for 4 h on a Multi Reax shaker (Heidolph Instruments, Schwabach, Germany) set to 1200 rpm. After extraction, the hot suspensions were centrifuged at 4350 rpm for 2 min, and the resulting supernatants were filtered through 0.22 µm Acrodisc PSF GHP syringe filters (Pall Life Sciences, New York, NY, USA). Chromatographic separation of carbohydrates was performed on a Sugar-Pak I cation-exchange column (6.5 × 300 mm, 10 µm; Waters, Milford, CT, USA), equipped with a Sugar-Pak Guard-Pak cartridge, and maintained at 90 °C. Isocratic elution with deionized water was applied at a flow rate of 0.6 mL/min, using a 10 µL injection volume. The total run time was 20 min [79]. External calibration curves for sucrose, D-glucose, and D-fructose (Sigma-Aldrich, Steinheim, Germany) were constructed in the range 0–5000 µg/mL. Each sample was analyzed in triplicate.

### 3.4. Microbiological Analysis

A 10 g sample of red bell pepper was mixed with 90 mL of 0.85% NaCl solution and homogenized for 30 s using a Stomacher 400 Circulator (Cambridge, UK). The total viable count (TVC) was determined on plate count agar (PCA) following incubation at 30 °C for 72 h. Yeasts and molds (TYM) were enumerated on Dichloran Rose-Bengal Chloramphenicol (DRBC) agar after incubation at 25 °C for 120 h. Microbial colonies were quantified using a ProtoCOL 3 system (Synbiosis, Frederick, MD, USA), and results were expressed as log CFU/g. All analyses were performed in triplicate. All microbiological media were obtained from Oxoid Deutschland GmbH (Wesel, Germany) [80].

### 3.5. Structural and Molecular Analysis

To preserve samples for structural and molecular analyses (SEM, µCT, FTIR and TGA), fresh and pretreated pepper material was freeze-dried. The material was shock-frozen at −40 °C for 4 h (Shock Freezer HCM 51.20, Irinox, Conegliano, Italy) and then lyophilized in a laboratory freeze dryer (Gamma 1-16 LSC, Martin Christ, Osterode am Harz, Germany) at a shelf temperature of 30 °C for 48 h and a pressure of 0.630 mbar; the condenser temperature was maintained at −55 °C. After freeze-drying, samples were stored in high-barrier packaging at controlled room temperature until analysis.

#### 3.5.1. Structural Analysis (SEM, Micro-CT)

Red bell pepper microstructure was examined using scanning electron microscopy (SEM) and X-ray microcomputed tomography (µCT). For SEM analysis, dried pepper samples were affixed to aluminum stubs with double-sided carbon adhesive tabs and coated with a thin (~5 nm) gold layer in a sputter coater (EM ACE200, Leica, Vienna, Austria). The observations were performed in a Phenom XL scanning electron microscope (Thermo Fisher Scientific, Waltham, MA, USA) operated at an accelerating voltage of 10 kV under low-vacuum conditions (~10 Pa). For each treatment, at least four cross-sectional micrographs were acquired at 200× magnification to visualize cell wall integrity, voids, and fracture patterns [78].

Three-dimensional imaging of the internal architecture was performed using µCT. Pieces of pepper pericarp (approximately 2 × 3 cm) were fixed on a 25 mm sample holder and scanned in a SkyScan 1272 desktop microtomograph (Bruker microCT, Kontich, Belgium). Scans were acquired at 40 kV and 193 µA with a rotation step of 0.3° over 180°, yielding an isotropic voxel size of 13.3 µm. The projection data were reconstructed using NRecon (Bruker) and subsequently segmented and quantified in CTAn (Bruker) to obtain binarised datasets [81].

#### 3.5.2. Time Domain Nuclear Magnetic Resonance (TD-NMR)

Water mobility and distribution in red bell pepper tissue were characterized by time-domain nuclear magnetic resonance (TD-NMR). Measurements were performed on a low-field NMR analyzer operating at a proton resonance frequency of approximately 20 MHz, equipped with a temperature-controlled probe. Cylindrical pieces of pepper tissue were cut to fit the NMR tube, carefully placed in the detection coil and equilibrated at 25 °C prior to analysis. Transverse relaxation (T_2_) was recorded using a Carr–Purcell–Meiboom–Gill (CPMG) pulse sequence. A 90–180° pulse arrangement was applied with an echo time (τ) selected to minimize instrumental dead time and susceptibility artifacts. For each sample, several thousand echoes were collected, and 16–32 scans were accumulated to improve the signal-to-noise ratio, with a recycle delay long enough to allow full relaxation of the longitudinal magnetization. The decay curves of the CPMG signal were fitted by inverse Laplace transformation to obtain T_2_ relaxation time distributions [82]. The resulting peaks were interpreted as proton populations associated with differently bound water fractions in the tissue (e.g., tightly bound, intra-cellular and extra-cellular water). Changes in the relative contribution and position of the T_2_ components were used to assess the effect of PEF treatment on water status and microstructural integrity of red bell pepper.

#### 3.5.3. Fourier-Transform Infrared Spectroscopy (FTIR)

Molecular-level changes in the pepper tissue were analyzed by Fourier-transform infrared spectroscopy using a Cary 630 FTIR spectrometer (Agilent Technologies, Santa Clara, CA, USA) equipped with a single-bounce diamond attenuated total reflectance (ATR) accessory. Dried, ground samples were placed directly on the ATR crystal and gently pressed to ensure good contact with the surface. For each sample, spectra were collected in the range 4000–650 cm^−1^ with a spectral resolution of 4 cm^−1^, and 64 scans were co-added to enhance the signal-to-noise ratio [83]. After each measurement, a fresh background spectrum was recorded, and the ATR crystal was cleaned with isopropanol. Spectral acquisition and basic processing (e.g., baseline correction, normalization) were carried out in MicroLab FTIR software version 1.1.13 (Agilent). Each sample was measured in triplicate, and the averaged spectrum was used for further qualitative analysis.

#### 3.5.4. Thermogravimetric Analysis (TGA)

The thermal stability and decomposition behavior of freeze-dried pepper samples were evaluated by thermogravimetric analysis using a TGA/DSC 3+ instrument (Mettler Toledo, Greifensee, Switzerland). Approximately 6 mg of dried material was weighed into 70 µL alumina crucibles. The samples were heated from 30 to 600 °C at a constant rate of 5 °C min^−1^ under a nitrogen flow of 50 mL min^−1^ to ensure an inert atmosphere [84].

During the experiment, mass loss (TG) and the rate of mass change (DTG) were continuously recorded. The TG/DTG curves were subsequently used to distinguish individual stages of thermal decomposition and to determine characteristic temperatures corresponding to maximum degradation rates. Data processing and evaluation were performed using STARe Evaluation Software (version 16.10, Mettler Toledo).

### 3.6. Statistical Analysis

All experiments were performed in triplicate (n = 3). Data were analyzed using one-way analysis of variance (ANOVA), followed by Tukey’s post hoc test to determine significant differences between treatment groups. Differences were considered statistically significant at *p* < 0.05. Normality and homogeneity of variance were verified prior to analysis. Pearson’s correlation analysis was used to identify relationships between physicochemical, bioactive, and antioxidant parameters. Principal component analysis (PCA) was conducted to visualize the overall variability among samples according to the applied PEF energy levels. PCA was performed using standardized data. All variables were mean-centered and scaled to unit variance prior to analysis to minimize the influence of different measurement units. The following variables were included in the PCA model: cell disintegration index (CDI), total phenolic content (TPC), total flavonoid content (TFC), antioxidant activities (ABTS, DPPH, FRAP), vitamin C, sucrose, glucose and fructose contents, total carotenoid content (TCC), color parameters of the external and internal (L*, a*, b*) tissue, and texture parameter (hardness). For grouping patterns and similarities among samples, hierarchical cluster analysis (HCA) was performed using Ward’s method and Euclidean distance. Statistical significance was *p* < 0.05 for all tests.

## 4. Conclusions

Pulsed electric field (PEF) treatment induced energy-dependent changes in the physicochemical and structural properties of red bell pepper. Low energy inputs (1–2 kJ/kg) resulted in mild and largely reversible electroporation, which slightly reduced firmness while preserving overall tissue integrity. Intermediate energy levels (4–5 kJ/kg) promoted irreversible electroporation, as reflected by a significant increase in the cell disintegration index CDI and the development of a more porous microstructure. These conditions enhanced the extractability of carotenoids and vitamin C without causing excessive structural collapse. In contrast, the highest applied energy level (10 kJ/kg) led to pronounced tissue disintegration, substantial softening, and marked alterations in color, indicating the onset of quality deterioration. Importantly, PEF treatment also contributed to improved microbial safety, as evidenced by the reduction in total viable counts and yeast and mold counts, particularly at higher energy inputs.

From an industrial perspective, the results indicate that moderate PEF treatment can be effectively applied as a non-thermal pre-treatment to improve mass transfer-dependent operations, such as drying, freezing, juice extraction, and the recovery of bioactive compounds. PEF energies in the range of 4–5 kJ/kg appear to offer a favorable balance between enhanced release of valuable compounds and acceptable texture preservation, making this approach attractive to produce minimally processed, high-quality vegetable products. However, this study has several limitations, e.g., only one electric field strength was investigated. Additionally, the experiments were conducted on a single cultivar and with uniform sample geometry, which may limit the direct transferability of the results to industrial-scale operations. Future research should focus on the optimization of PEF parameters across different field strengths, pulse widths, and product geometries. Further work should also investigate the long-term storage stability and sensory properties of PEF-treated products to fully assess their commercial potential.

## Figures and Tables

**Table 1:** Total color difference (ΔE) and texture properties (hardness) of red bell pepper tissue after PEF treatment with applied different energies.

Sample Code	Total Color Difference ΔE [-]		Hardness [N]
	External Tissue (Peel)	Internal Tissue	
F	-	-	52.3 ± 4.52 a *
PEF1	3.25 ± 1.25 c	14.8 ± 1.69 a	41.6 ± 3.57 b
PEF2	6.59 ± 1.76 b	10.0 ± 1.52 cd	43.3 ± 3.75 b
PEF4	5.38 ± 0.97 bc	10.8 ± 1.38 bc	38.3 ± 2.89 b
PEF5	4.24 ± 1.17 bc	13.7 ± 0.93 ab	39.2 ± 4.25 b
PEF10	15.6 ± 1.84 a	7.6 ± 2.79 d	38.9 ± 3.98 b

* a–d—different letters in the same column indicate different homogeneous groups (α = 0.05).

**Table 2:** Bioactive compounds (TPC, TFC, vitamin C, TCC) of red bell pepper after PEF treatment with applied different energies.

Sample Code	TPC [mg Chlorogenic Acid/100 g d.m.]	TFC [mg QE/100 g d.m.]	Vitamin C [mg/100 g d.m.]	TCC [mg β-Carotene/100 g d.m.]
F	4152 ± 210 a *	452 ± 28 a	2099 ± 85 ab	1264 ± 38 c
PEF1	3933 ± 93 ab	360 ± 17 b	2325 ± 158 a	1310 ± 50 bc
PEF2	3906 ± 104 ab	326 ± 7 bcd	2130 ± 150 ab	1325 ± 76 abc
PEF4	3698 ± 176 b	292 ± 6 d	2009 ± 60 b	1456 ± 53 a
PEF5	3729 ± 61 b	352 ± 12 bc	2173 ± 90 ab	1413 ± 31 ab
PEF10	3617 ± 37 b	318 ± 4 cd	2232 ± 57 ab	1209 ± 37 c

* a–d—different letters in the same column indicate different homogeneous groups (α = 0.05).

**Table 3:** Antioxidant activity (ABTS, DPPH, FRAP assay) of red bell pepper after PEF treatment with applied different energies.

Sample Code	ABTS [mg TE/g d.m.]	DPPH [mg TE/g d.m.]	FRAP [mg TE/g d.m.]
F	17.1 ± 0.66 a *	29.4 ± 0.86 a	43.0 ± 1.52 a
PEF1	14.3 ± 0.53 a	20.2 ± 1.32 b	28.0 ± 0.48 cd
PEF2	15.6 ± 0.46 ab	21.8 ± 0.64 b	30.4 ± 1.34 bc
PEF4	17.1 ± 0.74 c	19.3 ± 1.23 b	31.6 ± 1.10 b
PEF5	17.7 ± 0.94 ab	20.5 ± 0.28 b	31.4 ± 0.74 b
PEF10	17.6 ± 0.73 bc	20.9 ± 1.67 b	25.1 ± 0.76 d

* a–d—different letters in the same column indicate different homogeneous groups (α = 0.05).

**Table 4:** Sugars content after PEF treatment with applied different energies.

Sample Code	Sucrose [g/100 g d.m.]	Glucose [g/100 g d.m.]	Fructose [g/100 g d.m.]
F	0.73 ± 0.15 b *	24.6± 1.22 bc	35.3 ± 0.73 a
PEF1	1.08 ± 0.10 a	26.5 ± 1.05 ab	32.8 ± 2.74 a
PEF2	1.18 ± 0.05 a	27.3 ± 0.26 a	36.1 ± 0.76 a
PEF4	0.54 ± 0.09 b	24.4 ± 1.40 bc	33.7 ± 0.80 a
PEF5	0.53 ± 0.05 b	23.2 ± 0.57 c	27.9 ± 1.27 b
PEF10	0.27 ± 0.03 c	24.7 ± 0.19 bc	26.5 ± 2.50 b

* a–c—different letters in the same column indicate different homogeneous groups (α = 0.05).

**Table 5:** Effect of PEF energy on mass loss and decomposition temperatures of red bell pepper in thermogravimetric analysis.

Sample Code	Step 1 (30–110 °C)		Step 2 (110–250 °C)		Step 3 (250–480 °C)		Step 4 (480–600 °C)	
	Mass Loss [%]	Decomposition Temperature [°C]	Mass Loss [%]	Decomposition Temperature [°C]	Mass Loss [%]	Decomposition Temperature [°C]	Mass Loss [%]	Decomposition Temperature [°C]
F	4.4	77	32.1	181	29.4	310	2.3	- *
PEF1	4.8	63	34.5	183	30.7	311	2.5	-
PEF2	4.8	64	34.4	182	29.4	310	2.4	-
PEF4	4.4	67	34.4	183	29.0	311	2.4	-
PEF5	3.9	68	35.1	184	29.3	311	2.5	-
PEF10	4.1	68	34.5	183	29.0	311	2.5	-

* no data.

**Table 6:** Description of samples and PEF treatment conditions.

Sample Code	Description	Specific Energy Input [kJ/kg]
F	Fresh, untreated sample (control)	0
PEF1 *	Pulsed Electric Field treated	1
PEF2	Pulsed Electric Field treated	2
PEF4	Pulsed Electric Field treated	4
PEF5	Pulsed Electric Field treated	5
PEF10	Pulsed Electric Field treated	10

* All samples were prepared from homogenized red bell pepper (*Capsicum annuum* L.) and processed under a temperature of 21.5 ± 0.2 °C.

## Data Availability

The original contributions presented in this study are included in the article and Supplementary Materials. Further inquiries can be directed to the corresponding author.

## Supplementary Materials

The following supporting information can be downloaded at https://www.mdpi.com/article/10.3390/molecules31010088/s1: Table S1: Pearson’s correlation coefficients of red bell pepper tissue treated with different energies of PEF treatment.
