# Supplementary material for: Energy-Dependent Effects of Pulsed Electric Field (PEF) Treatment on the Quality Attributes, Bioactive Compounds, and Microstructure of Red Bell Pepper
Source: Molecules. 2025 Dec 25;31(1):88. doi: 10.3390/molecules31010088 (PMC12787053; doi:10.3390/molecules31010088)
Supplement: Supplementary file 1 [file molecules-31-00088-s001.zip › molecules-4022302-supplementary.pdf]

molecules

MDPI

Supplementary Materials

Table S1. Pearson's correlation coefficients of red bell pepper tissue treated with different energies of PEF treatment.

Parameter	CDI	TPC	TFC	ABTS	DPPH	FRAP	Vitamin C	Sucrose	Glucose	Fructose	TCC	L*_ex	a*_ex	b*_ex	L*_in	a*_in	b*_in	Hardness
CDI	1.0000	-.9801	-.8556	.4101	-.7959	-.7498	-.0272	-.3246	-.3237	-.6841	.2964	.8025	-.3360	-.1198	-.6320	-.5413	-.8158	-.9109
	p= ---	p=.001	p=.030	p=.419	p=.058	p=.086	p=.959	p=.530	p=.531	p=.134	p=.568	p=.055	p=.515	p=.821	p=.178	p=.267	p=.048	p=.012
TPC	-.9801	1.0000	.8504	-.4009	.8042	.7695	-.0162	.4158	.3593	.7240	-.2527	-.8212	.4200	.2274	.6695	.4855	.7625	.9263
	p=.001	p= ---	p=.032	p=.431	p=.054	p=.074	p=.976	p=.412	p=.484	p=.104	p=.629	p=.045	p=.407	p=.665	p=.146	p=.329	p=.078	p=.008
TFC	-.8556	.8504	1.0000	-.0135	.9010	.8046	.0842	-.0046	-.0860	.2891	-.3916	-.5205	.0702	-.1875	.7021	.0775	.7906	.9054
	p=.030	p=.032	p= ---	p=.980	p=.014	p=.054	p=.874	p=.993	p=.871	p=.578	p=.443	p=.290	p=.895	p=.722	p=.120	p=.884	p=.061	p=.013
ABTS	.4101	-.4009	-.0135	1.0000	.1674	.2184	-.5108	-.6896	-.8424	-.4927	.0763	.6278	-.4272	-.4112	.3553	-.6996	.0398	-.0785
	p=.419	p=.431	p=.980	p= ---	p=.751	p=.678	p=.300	p=.130	p=.035	p=.321	p=.886	p=.182	p=.398	p=.418	p=.489	p=.122	p=.940	p=.882
DPPH	-.7959	.8042	.9010	.1674	1.0000	.8705	-.2137	-.1352	-.0438	.4094	-.4607	-.3707	-.0249	-.1988	.9003	.1901	.9250	.9663
	p=.058	p=.054	p=.014	p=.751	p= ---	p=.024	p=.684	p=.798	p=.934	p=.420	p=.358	p=.469	p=.963	p=.706	p=.014	p=.718	p=.008	p=.002
FRAP	-.7498	.7695	.8046	.2184	.8705	1.0000	-.5146	.1189	-.2213	.5480	.0312	-.5653	.3537	.1539	.9409	.0705	.7656	.8573
	p=.086	p=.074	p=.054	p=.678	p=.024	p= ---	p=.296	p=.822	p=.673	p=.260	p=.953	p=.242	p=.492	p=.771	p=.005	p=.894	p=.076	p=.029
Vitamin C	-.0272	-.0162	.0842	-.5108	-.2137	-.5146	1.0000	-.0598	.3041	-.4172	-.5121	.0623	-.3501	-.4036	-.6118	.0509	-.1857	-.1479
	p=.959	p=.976	p=.874	p=.300	p=.684	p=.296	p= ---	p=.910	p=.558	p=.411	p=.299	p=.907	p=.496	p=.427	p=.197	p=.924	p=.725	p=.780
Sucrose	-.3246	.4158	-.0046	-.6896	-.1352	.1189	-.0598	1.0000	.5441	.6839	.5194	-.7911	.9292	.9210	-.0409	.3331	-.2041	.0919
	p=.530	p=.412	p=.993	p=.130	p=.798	p=.822	p=.910	p= ---	p=.264	p=.134	p=.291	p=.061	p=.007	p=.009	p=.939	p=.519	p=.698	p=.862
Glucose	-.3237	.3593	-.0860	-.8424	-.0438	-.2213	.3041	.5441	1.0000	.5830	-.2947	-.4021	.2535	.3617	-.1723	.8123	.0805	.1644
	p=.531	p=.484	p=.871	p=.035	p=.934	p=.673	p=.558	p=.264	p= ---	p=.225	p=.571	p=.429	p=.628	p=.481	p=.744	p=.050	p=.880	p=.756
Fructose	-.6841	.7240	.2891	-.4927	.4094	.5480	-.4172	.6839	.5830	1.0000	.1565	-.8039	.7018	.6743	.5193	.7203	.4717	.5841
	p=.134	p=.104	p=.578	p=.321	p=.420	p=.260	p=.411	p=.134	p=.225	p= ---	p=.767	p=.054	p=.120	p=.142	p=.291	p=.106	p=.345	p=.223
TCC	.2964	-.2527	-.3916	.0763	-.4607	.0312	-.5121	.5194	-.2947	.1565	1.0000	-.2471	.7107	.7236	-.1165	-.3064	-.5368	-.4177
	p=.568	p=.629	p=.443	p=.886	p=.358	p=.953	p=.299	p=.291	p=.571	p=.767	p= ---	p=.637	p=.113	p=.104	p=.826	p=.555	p=.272	p=.410
L*_ex	.8025	-.8212	-.5205	.6278	-.3707	-.5653	.0623	-.7911	-.4021	-.8039	-.2471	1.0000	-.8048	-.6324	-.3502	-.4839	-.3604	-.5706
	p=.055	p=.045	p=.290	p=.182	p=.469	p=.242	p=.907	p=.061	p=.429	p=.054	p=.637	p= ---	p=.053	p=.178	p=.496	p=.331	p=.483	p=.237

https://doi.org/10.3390/molecules31010088

a*_ex	-.3360	.4200	.0702	-.4272	-.0249	.3537	-.3501	.9292	.2535	.7018	.7107	-.8048	1.0000	.9490	.1762	.1785	-.1198	.1527
	p=.515	p=.407	p=.895	p=.398	p=.963	p=.492	p=.496	p=.007	p=.628	p=.120	p=.113	p=.053	p= ---	p=.004	p=.738	p=.735	p=.821	p=.773
b*_ex	-.1198	.2274	-.1875	-.4112	-.1988	.1539	-.4036	.9210	.3617	.6743	.7236	-.6324	.9490	1.0000	.0572	.2020	-.2806	-.0246
	p=.821	p=.665	p=.722	p=.418	p=.706	p=.771	p=.427	p=.009	p=.481	p=.142	p=.104	p=.178	p=.004	p= ---	p=.914	p=.701	p=.590	p=.963
L*_in	-.6320	.6695	.7021	.3553	.9003	.9409	-.6118	-.0409	-.1723	.5193	-.1165	-.3502	.1762	.0572	1.0000	.0969	.8026	.8485
	p=.178	p=.146	p=.120	p=.489	p=.014	p=.005	p=.197	p=.939	p=.744	p=.291	p=.826	p=.496	p=.738	p=.914	p= ---	p=.855	p=.055	p=.033
a*_in	-.5413	.4855	.0775	-.6996	.1901	.0705	.0509	.3331	.8123	.7203	-.3064	-.4839	.1785	.2020	.0969	1.0000	.4582	.3613
	p=.267	p=.329	p=.884	p=.122	p=.718	p=.894	p=.924	p=.519	p=.050	p=.106	p=.555	p=.331	p=.735	p=.701	p=.855	p= ---	p=.361	p=.482
b*_in	-.8158	.7625	.7906	.0398	.9250	.7656	-.1857	-.2041	.0805	.4717	-.5368	-.3604	-.1198	-.2806	.8026	.4582	1.0000	.9107
	p=.048	p=.078	p=.061	p=.940	p=.008	p=.076	p=.725	p=.698	p=.880	p=.345	p=.272	p=.483	p=.821	p=.590	p=.055	p=.361	p= ---	p=.012
